# Comparative Genomics of Two Newly Sequenced Rodent-Derived and One Previously Reported Tick-Derived *Borrelia garinii* Strains from South Korea Reveals Plasmid Variation and Virulence Gene Diversity

**DOI:** 10.3390/pathogens14111182

**Published:** 2025-11-18

**Authors:** Hyungsuk Kang, Yeon-Joo Choi, Ji-Young Park, Kwangjun Lee, Won-Jong Jang

**Affiliations:** 1 Department of Microbiology, Konkuk University School of Medicine, 120 Neungdong-ro, Gwangjin-gu, Seoul 05029, Republic of Korea; kupa88@kku.ac.kr (H.K.);; 2 Research Institute of Medical Science, Konkuk University School of Medicine, 120 Neungdong-ro, Gwangjin-gu, Seoul 05029, Republic of Korea; 3 Institute of Biomedical Sciences & Technology, Konkuk University, 120 Neungdong-ro, Gwangjin-gu, Seoul 05029, Republic of Korea; 4 Division of Zoonotic and Vector Borne Disease Research, National Institute of Health, Korea Disease Control and Prevention Agency, Osong, Cheongju 28160, Republic of Korea

**Keywords:** *Borrelia garinii*, whole-genome sequencing, tick-borne pathogen, lyme borreliosis

## Abstract

*Borrelia garinii* is a spirochete associated with Lyme borreliosis and is widely distributed across Eurasia. Although its genomic features have been well characterized in Europe, genomic data from East Asian isolates remain limited. Two *B. garinii* strains, HN13 and HN18, were isolated from a wild rodent (*Apodemus agrarius*) in South Korea and subjected to whole-genome sequencing and comparative genomic analysis. Their genomic features were compared with those of a tick-derived Korean strain 935 and additional global reference genomes. Phylogenetic analyses revealed that *B. garinii* strain HN18 clustered closely with French strains CIP103362 and 20047, whereas *B. garinii* strain HN13 showed high chromosomal similarity to the Korean strain 935. Both rodent-derived strains harbored plasmids carrying virulence-associated genes, including *vlsE* and *vls* silent cassettes, which were absent in *B. garinii* strain 935. This study provides new genomic insights into *B. garinii* circulating in East Asia and reveals host-associated plasmid variation linked to virulent potential. This study also suggests possible trans-Eurasian gene flow and underscores the need for continued genomic surveillance to better understand the evolution and epidemiology of *Borrelia* species.

## 1. Introduction

*Borrelia garinii* is a Gram-negative, microaerophilic, slowly growing, extracellular spirochete that belongs to the family Spirochaetaceae [1,2,3,4]. It is one of the main causative agents of Lyme borreliosis [5] and is transmitted primarily by Ixodes ticks that are known vectors of the pathogen [5,6]. The first isolation of *B. garinii* was reported in 1992 and named after the French physician ‘Charles Garin’ [7]. *B. garinii* is now known to be widely distributed across the Northern Hemisphere [8], which includes countries in Europe (e.g., Germany [9], France [10], England, the Czech Republic, Switzerland, Slovakia, Serbia, Finland [11], Italy [12], Austria [13], Russia [14], Denmark, Slovenia [15], Sweden [7], the Netherlands [16], and Estonia [17]); North America (USA [18] and Canada [19]); and Asia (China [20], Japan [21], Mongolia [22], and Taiwan [23]), including South Korea [24,25,26].

In South Korea, *B. garinii* was first reported in 1993 [27]. Since then, it has been detected in ticks [28,29], clinical samples [24], and wild rodents such as *Apodemus agrarius* [26]. However, national surveillance studies suggested that *Borrelia afzelii* is the dominant Lyme borreliosis species in South Korea, accounting for up to 62.5% of cases, while *B. garinii* comprises up to 3.7% [28,30,31].

*Borrelia garinii* has been associated with severe clinical manifestations, including skin lesions, arthritis and neuroborreliosis (e.g., meningitis, peripheral facial palsy) [2,32,33]. Its pathogenicity is partly attributed to its ability to evade the host immune system through mechanisms such as antigenic variation via the *vls* locus and modulation of outer surface proteins like OspC [2,34,35,36,37]. Among the genes involved in immune evasion and tissue adhesion, genes like *ospC* [38,39], *vlsE* [36,40], *dbpA* [41], and PFam54/60 [42] are commonly used as molecular markers for strain-specific virulence and antigenic variation.

The *Borrelia* genome comprises a highly conserved linear chromosome and a variable set of linear and circular plasmids that encode host-adaptive and virulence-associated genes [2,43,44]. Despite global efforts to characterize the genetic diversity of *B. garinii*, plasmid-borne virulence genes remain highly variable [2,45]. As of 2025, 172 whole-genome sequences (WGS) of *B. garinii* strains have been deposited in the NCBI, including one Korean strain (935) isolated from a tick (accession number: GCF_000714705.1) [25]. Despite the identification of *B. garinii* in ticks, rodents, and clinical samples in South Korea, high-quality whole-genome sequences from this region remain limited. Moreover, the extent of genomic variation, particularly at the plasmid level, and its relationship to host or vector origin is not well understood.

In this study, we conducted whole-genome sequencing of two *B. garinii* strains isolated from wild rodents (*A. agrarius*) in South Korea and performed comparative genomic analyses with a tick-derived Korean strain (935) and other globally reported *B. garinii* genomes. Our analysis focused on genome-wide phylogenetic placement, plasmid structural variation, and the distribution of virulence-associated genes to investigate host-associated genomic diversity among *B. garinii* strains.

## 2. Materials and Methods

### 2.1. Bacterial Sample Preparation

Two *B. garinii* strains were isolated from a rodent species (*A. agrarius*) captured in the Haenam region (latitude: 39.1543, longitude: 127.4457) of South Korea. The spirochetes were cultured using heart tissue from an individual rodent in BSK II medium at 32 °C under microaerophilic conditions [46]. Species identification was performed using multilocus sequence typing (MLST) targeting 8 housekeeping genes: *clpA*, *clpX*, *nifS*, *pepX*, *pyrG*, *recG*, *rplB* and *uvrA* [26]. The isolates were designated *B. garinii* strain HN13 and *B. garinii* strain HN18.

Genomic DNA was extracted and purified by Macrogen Inc. (Humanizing Genomics Macrogen, Seoul, Republic of Korea) using a commercially available DNA extraction kit (Wizard^®^ HMW DNA Extraction Kit, Promega, Fitchburg, WI, USA). The concentration of the extracted genomic DNA from *B. garinii* strain HN13 was 71.66 ng/µL in an elution volume of 164 µL with an average fragment size of 17,507 bp, whereas that from *B. garinii* strain HN18 was 41.11 ng/µL in 100 µL with an average fragment size of 39,545 bp. The A260/A280 ratios for both samples ranged between 1.8 and 2.0.

### 2.2. Whole-Genome Sequencing, Assembly, and Annotation

A hybrid sequencing approach was used, combining long-read and short-read platforms. Long-read sequencing was performed using the Revio system (Pacific Biosciences, Menlo Park, CA, USA), and short-read sequencing was performed with the NovaSeq X series (Illumina, San Diego, CA, USA). Long-read libraries were prepared using the commercially available kits, SMRTbell prep kit 3.0 (Pacific Biosciences, Menlo Park, CA, USA), with DNA concentration of 10 ng/µL and fragment sizes between 5000 bp and 20,000 bp. Short-read libraries were prepared using the TruSeq DNA Nano Kit (Illumina, San Diego, CA, USA), with average fragment sizes of 654 bp (HN18) and 625 bp (HN13).

PacBio HiFi reads were assembled using the Hifiasm 0.19.9 tool [47] with the default settings. Contigs shorter than 1000 bp were discarded. Error correction was performed using short Illumina reads with Inspector v1.0.1 [48]. Illumina reads were filtered at a Phred score of 30 or higher and trimmed using the Trimmomatic 0.38 tool [49]. Three rounds of polishing were conducted using the Pilon 1.22 tool [50].

Genome completeness was computed via the BUSCO 5.1.3 tool [51] with the bacterial ortholog database version 10 (Bacteria_odb10). Gene prediction and functional annotation were performed using the Prokka 1.14.6 tool [52]. Protein domain annotation was completed using InterProScan 5.34–73.0 [53] and PSI-BLAST 2.14.0 [54] with the EggNOG v4.5 database [55].

### 2.3. Comparative Genomic Analysis of Korean B. garinii Strains and Other B. garinii Strains

The assembled genomes of *B. garinii* strains HN13 and HN18 were compared with the previously registered Korean strain *B. garinii* strain 935 (accession number: GCF_000714705.1), which was isolated from *Ixodes persulcatus* [25]. In addition, 14 publicly available *B. garinii* WGS were retrieved from the NCBI database, filtered to include only assemblies at the ‘chromosome’ or ‘complete genome’ level. Genomes flagged as suppressed by RefSeq due to annotation failure were excluded.

Plasmid types of *B. garinii* strains HN13, HN18 and 935 were identified by BLAST (BLAST+ version 2.17.0; BLASTDB v5 database format) [54] searches against full-length contigs and gene annotations, combined with results from MOB-recon v3.1.9 implemented in the MOB-suite [56].

Phylogenetic relationships among the 17 *B. garinii* genomes were inferred based on core-genome single-nucleotide polymorphisms (SNPs) using chromosome sequences on the Galaxy web-based platform (https://usegalaxy.org/) [57]. For core-genome alignment, Prokka-generated ‘.gff’ files were used as input for Roary [58] with a minimum BLASTP identity threshold of 95% and a core gene definition of presence in 99% of genomes, resulting in 775 core genes. Recombination was identified and masked with Gubbins [59] using 5 iterations, and convergence was assessed using the weighted Robinson–Foulds metric. Analyses were run without an outgroup (unrooted), and the recombination-filtered alignment generated by Gubbins was used for subsequent tree inference. The maximum-likelihood (ML) phylogeny was reconstructed in IQ-TREE v2.4.0 [60] under the GTR + F+ASC + R2 model, with 1000 nonparametric bootstrap replicates performed to assess branch support. A consensus tree was generated from all bootstrap replicates, which showed an identical topology to the ML tree (Robinson–Foulds distance = 0). The resulting tree was visualized in MEGA12 [61].

As *ospC* and multilocus sequence typing (MLST) sequence types (STs) are commonly employed for *Borrelia* strain typing [62], *ospC* gene sequences from the 17 genomes were obtained from EggNOG annotation results and the NCBI WGS database [63]. MLST allelic profiles were determined using eight housekeeping genes, *nifS*, *clpA*, *rplB*, *pyrG*, *recG*, *clpX*, *pepX*, and *uvrA*, through the PubMLST *Borrelia* spp. database [64]. The *ospC* and concatenated MLST nucleotide sequences were aligned using the MUSCLE algorithm implemented in MEGA12, and ML phylogenetic trees were constructed with 1000 bootstrap replicates to assess branch support.

Whole-genome average nucleotide identity (ANI) values among the 17 genomes were calculated using FastANI v1.3 [65] in the Galaxy platform, and the resulting heatmap was visualized in Rstudio v2025.09.2 [66] using ggplot2 v3.5.2 [67] and reshape2 v1.4.4 [68].

The *ospC*-containing plasmids (cp26) and *vls* locus-containing plasmids (lp28 and lp36 types) were aligned using MAFFT v7.526 [69]. Because cp26 is circular, sequence ends were manually inspected and, when necessary, reverse-complemented to ensure proper orientation and alignment. Once terminal regions were corrected, the sequences were re-aligned with MAFFT, and a phylogenetic tree was constructed in IQ-TREE with 1000 bootstrap replicates and visualized in MEGA12.

The *vls* locus (*vlsE* and/or *vls* cassette) was identified using Prokka-generated ‘.gff’, ‘.ffn’, and ‘.faa’ files in conjunction with EggNOG and InterProScan results. The *vls* loci of *B. garinii* strains HN13 and HN18 were visualized using Prokka-generated ‘.genbank’ files, and their synteny plots were produced with Easyfig v. 2.2.5 [70].

## 3. Results

### 3.1. General Genomic Features of the Three Korean B. garinii Strains HN13, HN18, and 935

The general genome characteristics of the two newly isolated *B. garinii* strains HN13 and HN18 and the previously reported *B. garinii* strain 935 are summarized in Table 1. The total genome sizes were 1191,250 bp for *B. garinii* strain HN13, 1140,642 bp for HN18, and 1176,739 bp for 935, with a consistent chromosomal GC content of 28.4%. Although *B. garinii* strain HN13 had the largest total genome size, the chromosomal sequence of *B. garinii* strain 935 (918,136 bp) was slightly longer than those of HN13 (906,429 bp) and HN18 (906,038 bp), suggesting subtle differences.

All three strains harbored both linear and circular plasmids. *B. garinii* strains HN13 and HN18 shared 8 plasmids, including lp54, lp32-10, and lp28-4, whereas *B. garinii* strain 935 contained a fused plasmid, lp54_lp32-10, and lacked lp28-4. The number of predicted coding sequences (CDSs) was highest in *B. garinii* strain HN18 (1360), followed by *B. garinii* strain 935 (1332) and *B. garinii* strain HN13 (1312). All strains carried 3 rRNA and 32 tRNA genes.

### 3.2. Phylogeny and Comparative Analysis

**Figure 1:**
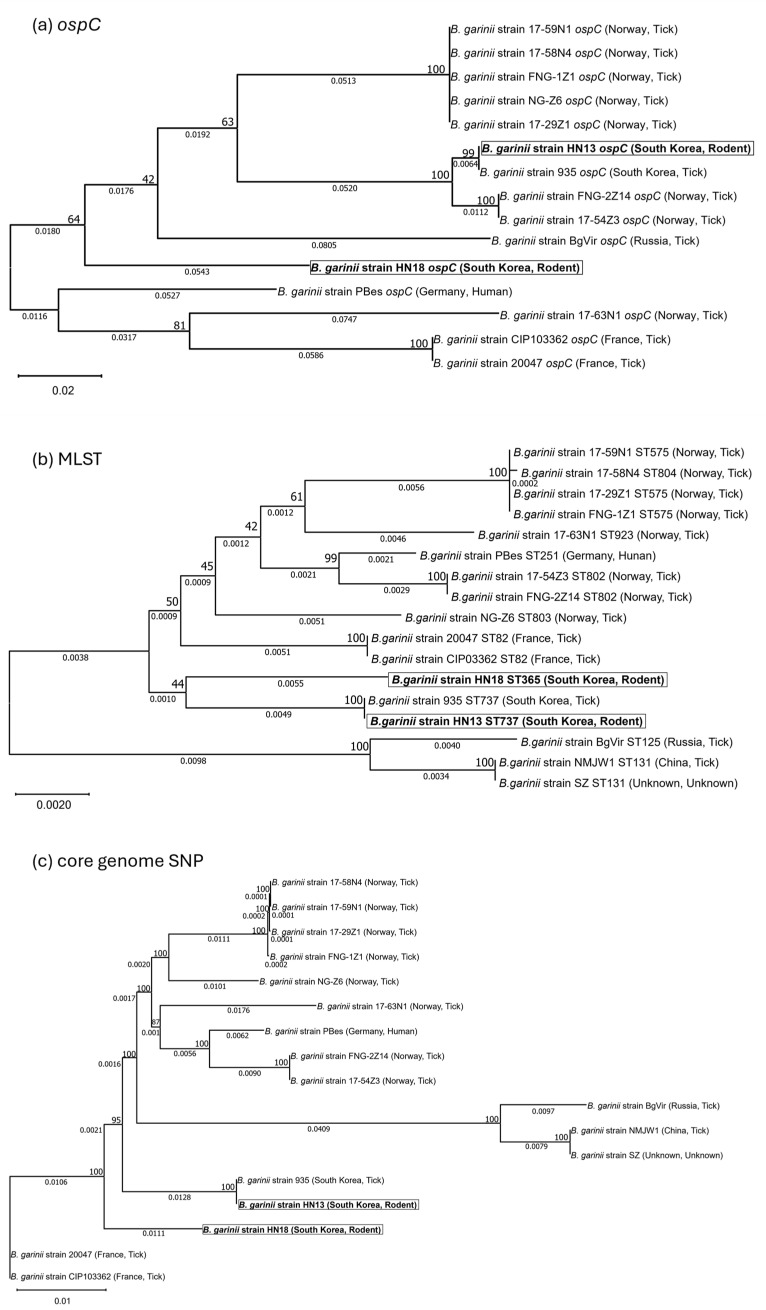
Phylogenetic relationships of Korean *B. garinii* strains based on (**a**) *ospC* gene sequences, (**b**) multilocus sequence typing (MLST), and (**c**) core-genome SNPs. Phylogenetic trees were constructed using the maximum-likelihood method in MEGA12 for (**a**,**b**) and in IQ-TREE for (**c**) with 1000 bootstrap replicates. Bootstrap support values are indicated at the nodes, and scale bars represent the number of substitutions per site. Novel strains HN13 and HN18 are highlighted in bold.

The phylogenetic relationships among *B. garinii* strains are shown in Figure 1. Based on *ospC*-based phylogenetic analysis (Figure 1a), *B. garinii* strain HN13 was identical (100% sequence similarity) to *B. garinii* strain 935, while *B. garinii* strain HN18 formed a distinct branch. Most Norwegian strains clustered together, except for the *B. garinii* strain 17-63N1, while the South Korean and Russian strains were broadly grouped in the same cluster. In contrast, the German and French strains formed separate clusters. Notably, *B. garinii* strains SZ and NMJW1 lacked plasmids, and the *ospC* gene was also absent in these strains.

In the MLST-based phylogenetic analysis (Figure 1b), the three Korean strains clustered closely together, with *B. garinii* strains HN13 and 935 again showing 100% sequence identity, whereas *B. garinii* strain HN18 formed an independent branch. In this tree, Chinese and Russian strains were closely grouped, while the European strains formed a broad cluster that also included the three Korean strains.

The core-genome SNP (chromosomal) phylogeny (Figure 1c) showed a similar overall pattern; *B. garinii* strains HN13 and 935 were again 100% core-genome sequence identity, while *B. garinii* strain HN18 remained distinct from the other two Korean strains. The Chinese and Russian strains were again grouped together, consistent with the MLST-based tree, whereas the European strains clustered primarily according to their geographic origin.

These phylogenetic patterns suggest that the rodent-derived Korean strain HN13 likely shares a recent common ancestor with 935 or has undergone limited diversification, consistent with local clonal expansion within South Korea. In contrast, *B. garinii* strain HN18, although also isolated from a rodent in the same region, formed an independent lineage, implying either a distinct evolutionary origin or possible gene flow with non-Korean populations. The clustering of Chinese and Russian strains together, as well as the partial overlap of European isolates, reflects the complex geographic distribution of *B. garinii* and may indicate multiple introduction or migration events across Eurasia.

**Figure 2:**
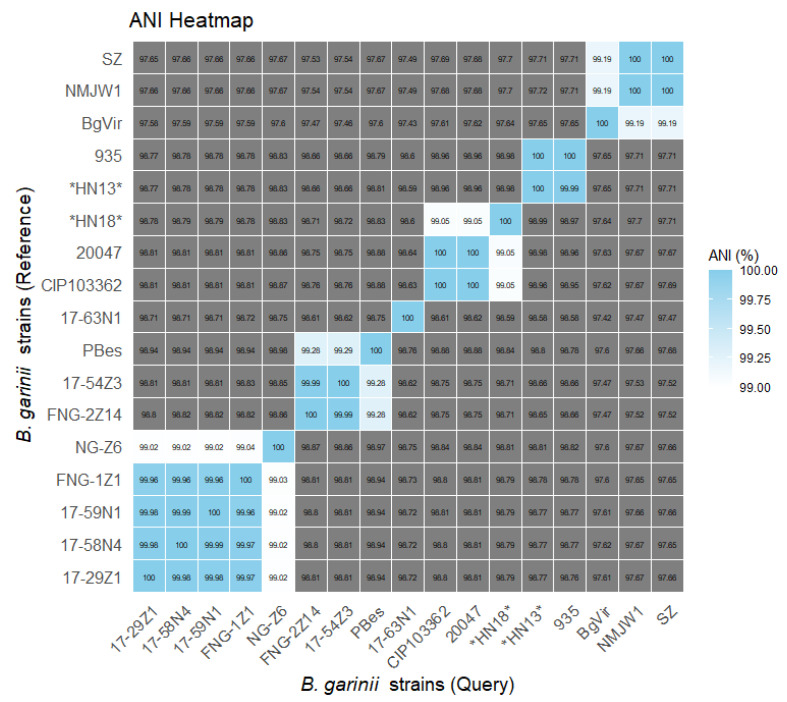
Average nucleotide identity (ANI) heatmap of 17 *B. garinii* chromosome sequences, including *B. garinii* strains HN13 and HN18 isolated in this study. The color scale indicates ANI values among the genomes, with gray shading representing ANI values below 99%. Novel strains HN13 and HN18 are highlighted with *.

The ANI analysis showed consistent results (Figure 2). *Borrelia garinii* strains HN13 and 935 exhibited nearly 100% chromosomal sequence identity, suggesting clonal or recently diverged origin within South Korea. In contrast, the *B. garinii* strain HN18 showed higher genomic similarity to the French strains CIP103362 and 20047, indicating a potential evolutionary connection between East Asian and European lineages. These patterns suggest that multiple *B. garinii* lineages, possibly representing both locally maintained and introduced strains, may coexist in the Korean Peninsula. The Chinese and Russian strains again formed a distinct cluster, and the Norwegian strains were also grouped together but separated into two subclusters.

### 3.3. Plasmid Content, Virulence Gene Distribution, and Comparative Analysis

The plasmid compositions of the 17 *B. garinii* strains are summarized in Table 2. Except for *B. garinii* strains NMJW1 and SZ, which lacked any plasmid sequences, all strains contained both lp54 and cp26. In addition, five strains possessed multiple lp28-type plasmids.

It is common for most *B. garinii* strains to possess the plasmids lp54, cp26, and multiple lp28s, which harbor several virulence-related genes. Specifically, *ospA*, *ospB*, *dbpA*, and *dbpB* were consistently located on the lp54 plasmid, whereas *ospC* was encoded on the cp26 plasmid. The *vlsE* gene and multiple *vls* silent cassettes were mainly detected on lp28-type plasmids (Table 3).

Among the three Korean strains, only *B. garinii* strains HN13 and HN18 carried the *vlsE* gene and multiple *vls* silent cassettes, both located on plasmid lp28-3 (Supplementary Table S1). *Borrelia garinii* strain HN13 additionally possessed one *vls* cassette on lp28-4.

Across all 17 *B. garinii* strains analyzed in this study, only 6 strains—two Korean strains, HN13 and HN18; two French strains, 20047 and CIP103362; a German strain, Pbes; and a Norwegian strain, 17-63N1—harbored *vlsE* and at least one *vls* silent cassette, primarily on lp28-type plasmids, although some French strains carried them on plasmid lp36.

**Figure 3:**
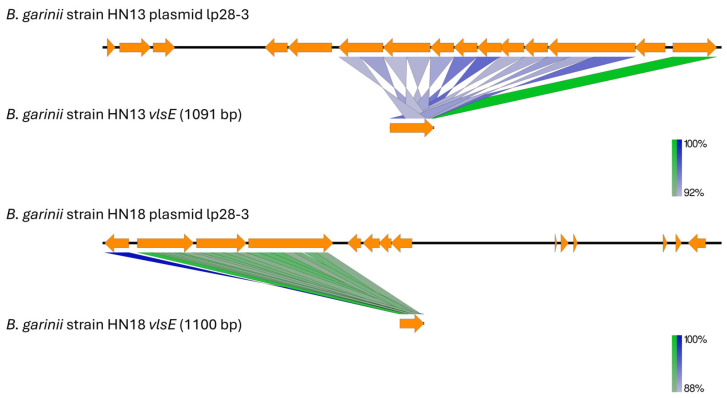
Comparison of plasmid p28-3 from *B. garinii* strains HN13 and HN18 showing the locations of *vlsE* and *vls* silent cassettes. Green regions indicate alignments in the same orientation, whereas blue regions indicate alignments in the opposite orientation. The *vlsE* gene (1091 bp in *B. garinii* strain HN13 and 1100 bp in HN18) and adjacent *vls* silent cassettes are located within the lp28-3 plasmid of both strains. The comparison was visualized using Easyfig v.2.2.5 [70].

The *vlsE*-carrying lp28-3 plasmids in *B. garinii* strains HN13 and HN18 also contained multiple *vls* silent cassettes (Figure 3). The *vlsE* gene was located near the 5’ end of the plasmid and was oriented in the opposite transcriptional direction to the downstream *vls* silent cassettes, highlighting the structural polarity of the *vls* locus.

**Figure 4:**
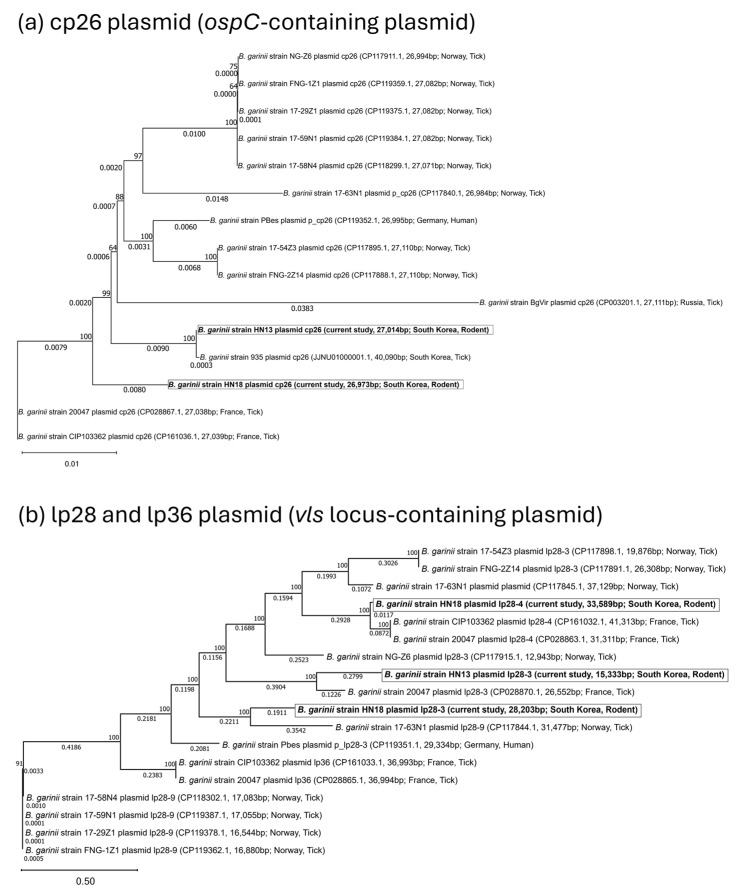
Phylogenetic analysis of plasmids containing *ospC* and *vls* loci in *Borrelia garinii*. (**a**) Maximum-likelihood phylogenetic tree of the cp26 plasmid (*ospC*-containing plasmid) constructed using the full nucleotide sequence of cp26 from available *B. garinii* strains. (**b**) Maximum-likelihood phylogenetic tree of plasmids carrying the *vls* locus, including lp28 and lp38 plasmid types. Trees were generated using MEGA 12 [61] with 1000 bootstrap replicates, and bootstrap values are shown at each node. Branch lengths represent the number of substitutions per site. *B. garinii* strain 13 and strain 18 from the current study are highlighted in bold.

Two phylogenetic trees based on cp26 plasmid (*ospC*-containing plasmid) and lp28 and lp36 plasmid types (*vls* locus-containing plasmid) are shown in Figure 4. The phylogenetic tree constructed from the cp26 plasmid (Figure 4a), which encodes *ospC*, exhibited a circular topology and showed a high degree of similarity to both the *ospC*-based phylogenetic tree (Figure 1a) and the core-genome SNP-based tree (Figure 1c). The two Korean strains HN13 and 935 clustered together, whereas *B. garinii* strain HN18 formed a distinct branch. The French strains CIP103362 and 20047 showed 100% sequence identity in cp26, while the Russian strain BgVir again formed a long, separate branch, consistent with the *ospC* and core-genome SNP-based phylogenies (Figure 1a,c). This phylogenetic concordance indicates that the evolutionary trajectories of the cp26 plasmid and its encoded *ospC* gene are highly linked, potentially reflecting co-inheritance or selective pressures that maintain plasmid-gene linkage. Such consistency across plasmid- and chromosome-based trees may indicate that the cp26 replicon contributes to the evolutionary diversification and ecological adaptation of *B. garinii* lineages.

A similar pattern was observed for the *vls* locus-containing plasmids (lp28 and lp36 types) (Figure 4b). In contrast to the cp26 tree, the two Korean strains HN13 and HN18 clustered closely with the French strains 20047 and CIP103362, as well as the Norwegian strain 17-63N1 lp28-9, which also carries the *vlsE* gene. However, the Korean strains formed their own distinct plasmid branches, whereas the French strains again shared 100% identical plasmid sequences in cp26, lp28-4 and lp36. Similarly, two Norwegian strains (17-54Z3 and FNG-2Z14) showed 100% identity in cp26 and lp28-3. These findings suggest that although the Korean strains share general plasmid similarity with European lineages, their plasmids have diverged into separate, lineage-specific variants rather than remaining 99–100% identical, implying independent circulation and local evolutionary differentiation.

## 4. Discussion

In this study, two *B. garinii* strains, HN13 and HN18, were isolated from wild rodents (*A. agrarius*) in South Korea and compared with the previously sequenced *B. garinii* strain 935, which was isolated from the tick *I. persulcatus* [25] in South Korea. Whole-genome analysis revealed that *B. garinii* strain HN13 shared high chromosomal similarity with 935, as supported by *ospC*, MLST, and core-genome SNP analyses. However, the two strains differed substantially in plasmid composition and virulent gene profiles.

*Borrelia garinii* strain HN13 possessed plasmids lp28-3 and lp28-4, both of which harbored *vlsE* and multiple *vls* silent cassettes. In contrast, *B. garinii* strain 935 lacked the *vlsE* system entirely, despite possessing plasmid lp28-3. In addition, *B. garinii* strain 935 carried a unique hybrid plasmid (lp54_lp32-10) and lacked lp28-4. These findings aligned with previous reports that demonstrate high conservation in *Borrelia* chromosomes but extensive plasmid variability in Borrelia, particularly in plasmids carrying virulence determinants [43,44,71]. Loss of virulence-associated plasmids, including the absence of *vlsE*, has been associated with reduced infectivity and adaptation to non-vertebrate hosts such as ticks [40,72]. Although *B. garinii* is widespread across East Asia [73,74], most surveillance studies have been performed at the species level rather than at the strain level. Thus, this work represents a comparative genomic analysis of East Asian *B. garinii* strains and establishes a foundation for future evolutionary and epidemiological investigations.

Phylogenetic analysis of the *vlsE*-containing plasmids indicated that *B. garinii* strains HN13 and HN18 clustered closely with several European strains, including *B. garinii* strains 20047 and CIP103362 (France) and strain 17-63N1 (Norway). In contrast, the cp26 plasmid encoding *ospC* was conserved across all three Korean strains. The phylogenetic concordance between the cp26 plasmid and *ospC*-based trees further supports the notion that the evolution of cp26 is tightly linked to that of its encoded *ospC*, likely reflecting co-inheritance or selective pressures that maintain gene–plasmid compatibility.

Notably, *B. garinii* strain HN18 clustered with French *B. garinii* strains 20047 and CIP103362 across multiple analyses, including core-genome SNPs, ANI heatmap, and plasmid phylogeny, suggesting a potential historical connection between South Korean and French *B. garinii* populations. Combined with the near-identical chromosomal similarity between HN13 and 935, these findings indicate the coexistence of at least two distinct *B. garinii* lineages in South Korea: one locally maintained (HN13/935) and another genetically affiliated with French strains (HN18). A plausible mechanism for this connectivity is migratory birds, which serve as vertebrate hosts for *Ixodes* ticks across overlapping habitats in Europe and Asia [19,75,76,77,78,79]. For example, *B. garinii* has been detected in *Ixodes ricinus* on *Fringilla coelebs*, a bird species migrating between Europe, Central Asia, and western China (https://www.datazone.birdlife.org, accessed on 15 September 2025) [80], and in *Ixodes persulcatus* feeding on *Turdus naumanni*, which occurs across East Asia and the Korean Peninsula [81,82]. Rodents, including *A. agrarius*, are also known reservoirs for *Borrelia* species and likely maintain local transmission cycles [77,82].

The virulence gene profiles further support differential pathogenic potential among the strains. *Borrelia garinii* strain HN18 possessed a full complement of key virulence genes, including *ospA*, *ospB*, *dbpA*, *dbpB*, *vlsE*, and PFam54/60 family protein, suggesting a high virulence potential. *Borrelia garinii* strain HN13 also carried *vlsE* but lacked *dbpA*, whereas *B. garinii* strain 935 lacked both *vlsE* and *dbpA*. Because *vlsE* is associated with persistent infection and immune evasion [83,84], and *vlsE* and *dbpA* with tissue adhesion and Lyme arthritis [36,41], these genetic differences may have clinical relevance. Interestingly, all three Korean *B. garinii* strains encoded *ospC*, typically linked to the early stage of Lyme borreliosis [84], whereas only the rodent-derived strains (HN13 and HN18) carried *vlsE*, which is essential for long-term persistence in vertebrate hosts [83,84]. These findings support the hypothesis that 935 may have adapted to a non-vertebrate (tick) host and lost certain virulence traits.

In human Lyme borreliosis, *vlsE* expression promotes antigenic variation and immune evasion during chronic infection, contributing to disease persistence and treatment resistance [37,72]. Although clinical data for Korean *B. garinii* infections are not yet available, the presence of *vlsE*-harboring plasmids in rodent-derived strains suggests that such lineages may possess an enhanced capacity for persistent infection in vertebrate hosts. Comparative analyses with human-derived *B. garinii* isolates will be important to evaluate whether these virulence determinants correlate with clinical severity or treatment outcomes.

Despite the insights gained, the number of Korean *B. garinii* genomes available in public databases remains limited, restricting broader regional comparisons. To ensure analytical robustness, this study included only complete or chromosome-level assemblies with well-annotated plasmid structures, enabling accurate assessment of gene content and plasmid diversity. This approach revealed both conserved and lineage-specific genomic features among *B. garinii* strains from Europe, Russia, China and South Korea. Further sequencing of additional isolates from diverse hosts and geographic regions will be necessary to more fully resolve the evolutionary dynamics and pathogenic heterogeneity of *B. garinii* in East Asia.

The genomic differences observed among the Korean *B. garinii* strains also have potential epidemiological and public health relevance. The coexistence of rodent-derived *vlsE*-positive strains (HN13 and HN18) and a tick-adapted *vlsE*-negative strain (935) suggests the presence of multiple transmission cycles involving both vertebrate and arthropod hosts in the region. Such genomic heterogeneity may influence transmission dynamics, host range, and the risk of human exposure. From a public health perspective, the detection of *vlsE*-harboring plasmids in local rodent isolates underscores the need for continued surveillance to monitor the emergence of potentially more virulent *B. garinii* lineages and to strengthen early warning systems for Lyme borreliosis in East Asia.

## 5. Conclusions

This study provides the first comparative genomic analysis of rodent- and tick-derived *B. garinii* strains circulating in South Korea. Although *B. garinii* strains HN13 and 935 shared highly similar chromosomal sequences, they differed markedly in plasmid composition, and virulence gene profiles suggest host-associated adaptation within local transmission cycles. In contrast, the *B*. *garinii* strain HN18 represents a genetically distinct lineage that clustered closely with European *B. garinii* strains and carried a broader repertoire of virulence determinants. These findings highlight the coexistence of multiple *B. garinii* lineages in South Korea, including both locally maintained and potentially trans-Eurasian lineages, whose circulation may be shaped by migratory birds, rodents, and shared *Ixodes* vectors. Given the limited genomic data available from East Asia, continued genomic surveillance of *Borrelia* species across Eurasia is essential to better understand their evolutionary dynamics and assess their potential public health impact.

## Figures and Tables

**Table 1:** Genome assembly statistics and gene contents of three *Borrelia garinii* strains isolated in South Korea.

	*B. garinii* Strain HN13			*B. garinii* Strain HN18			*B. garinii* Strain 935 *		
Contigs	Size (bp)	GC (%)	Depth (x)	Size (bp)	GC (%)	Depth (x)	Size (bp)	GC (%)	Depth (x)
Chromosome	906,429	28.4	381	906,083	28.4	1251	918,136	28.4	-
lp54	52,486	26.9	2141	59,237	26.6	4813	-	-	-
lp36	25,219	23.6	1498	24,716	23.6	4286	40,674	23.9	-
cp32-6	29,083	29.1	1195	29,466	28.8	669	36,850	28.5	-
lp32-10	26,630	25.5	710	32,156	26.7	3690	-	-	-
lp28-3	15,333	33.8	391	28,203	31.4	1088	66,309	24.8	-
lp28-4	40,508	25.4	1033	33,589	25.1	2574	-	-	-
lp28-7	-	-	-	28,225	32.2	4472	-	-	-
cp26	27,014	25.9	1552	26,973	25.9	846	40,090	25.4	-
lp17	17,940	22.9	1582	22,602	24.2	6414	29,571	23.2	-
lp54_lp32-10	-	-	-	-	-	-	67,109	26.6	-

* Genome sequence information was obtained from GenBank (accession no.: GCF_000714705.1).

**Table 2:** Plasmid profiles of 17 *B. garinii* strains analyzed in this study.

Strains	Linear-Form Plasmids						Circular-Form Plasmids			Other *	Total
	lp54	lp36	lp32	lp28	lp25	lp17	cp32	cp26	cp9		
HN13	lp54	lp36	lp32-10	lp28-3 lp28-4	-	lp17	cp32-6	cp26	-	-	8
HN18	lp54	lp36	lp32-10	lp28-3 lp28-4 lp28-7	-	lp17	cp32-6	cp26	-	-	9
935	-	lp36	-	lp28-3	-	lp17	cp32-6	cp26	-	lp54_lp32-10	6
BgVir	lp54	-	-	-	-	-	-	cp26	-	-	2
20047	lp54	lp36	lp32-10	lp28-3 lp28-4 lp28-7	-	lp17	cp32-3 cp32-6	cp26	-	-	10
CIP103362	lp54	lp36	lp32-10	lp28-4 lp28-7	-	lp17	cp32-3 cp32-6	cp26	-	-	9
PBes	p_lp54	p_lp36	p_lp32-5 p_lp32-9 p_lp32-10	p_lp28-3 p_lp28-7	p_lp25	p_lp17	-	p_cp26	p_cp9	-	11
NMJW1	-	-	-	-	-	-	-	-	-	-	0
SZ	-	-	-	-	-	-	-	-	-	-	0
17-63N1	lp54	-	-	lp28-9	lp25	-	p_cp32-3 p_cp32-6	p_cp26	-	lp17_32-10 Plasmid	8
NG-Z6	lp54	-	-	lp28-2 lp28-3	lp25	lp17	-	cp26	cp9	Plasmid	8
FNG-1Z1	lp54	lp36	lp32-10	lp28-9	lp25	lp17	-	cp26	-	-	7
17-29Z1	lp54	lp36	lp32-10	lp28-9	lp25	lp17	-	cp26	-	-	7
17-54Z3	lp54	-	lp32-10	lp28-3	lp25	lp17	-	cp26	-	Plasmid	7
17-59N1	lp54	lp36	lp32-10	lp28-9	lp25	lp17	-	cp26	-	-	7
17-58N4	lp54	lp36	lp32-10	lp28-9	lp25	lp17	-	cp26	-	-	7
FNG-2Z14	lp54	-	lp32-10	lp28-3	lp25	lp17	-	cp26	-	-	6

* This column includes unnamed, unclassified, or combined plasmids in which more than two plasmid contigs were merged.

**Table 3:** Distribution of some of virulence-related genes identified on plasmids of 17 *Borrelia garinii* strains.

	*ospA*	*ospB*	*ospC*	*dbpA*	*dbpB*	*vlsE*/*vls* Silent Cassettes	PFam54/60 (Bbcrasp-1 Domain) **
HN13	lp54	lp54	cp26	-	lp54	lp28-3/lp28-3	lp54, lp36, lp32-10, lp28-4
HN18	lp54	-	cp26	lp54	lp54	lp28-3/lp28-3, lp28-4	lp54; lp36, lp32-10; lp28-4
935	lp54_lp32-10 *	lp54_lp32-10 *	cp26	-	lp54_lp32-10 *	-/-	lp54_lp32-10 *; lp36, lp28-3
BgVir	lp54	lp54	cp26	lp54	lp54	-/-	lp54
20047	lp54	-	cp26	lp54	lp54	lp36/lp36, lp28-3, lp28-4	lp54, lp36, lp32-10, lp28-4,
CIP103362	lp54	-	cp26	lp54	lp54	lp36/lp36, lp28-4	lp54, lp36, lp32-10, lp28-4,
PBes	p_lp54	-	p_cp26	-	p_lp54	p_lp28-3	p_lp54, p_lp32-10, p_lp28-3, p_lp28-5, p_lp25
NMJW1	No plasmid						
SZ	No plasmid						
17-63N1	lp54	-	p_cp26	lp54	lp54	lp28-9, plasmid	lp54, p_cp32-3, lp28-9, lp25, plasmid
NG-Z6	lp54	-	cp26	-	lp54	lp28-3	lp54, lp25, plasmid
FNG-1Z1	lp54	-	cp26	lp54	lp54	lp28-9	lp54, lp36, lp32-10, lp25
17-29Z1	lp54	-	cp26	lp54	lp54	lp28-9	lp54, lp36, lp32-10, lp25
17-54Z3	lp54	-	cp26	lp54	lp54	lp28-3	lp54, lp32-10, lp28-3, lp25
17-59N1	lp54	-	cp26	lp54	lp54	lp28-9	lp54, lp36, lp32-10, lp25
17-58N4	lp54	-	cp26	lp54	lp54	lp28-9	lp54, lp36, lp32-10, lp25
FNG-2Z14	lp54	-	cp26	lp54	lp54	lp28-3	lp54, lp32-10, lp28-3, lp25

* The virulence-associated genes were annotated in *B. garinii* strain 935 based on in silico analysis of the fused plasmid lp54_lp32-10. ** Pfam54/60: *Borrelia* Bbcrasp-1-domain-containing protein.

## Data Availability

The whole-genome sequencing datasets supporting this study have been deposited in the NCBI database under BioProject accessions PRJNA1250628 & PRJNA1250701 (BioSamples: SAMN47947845 & SAMN47947906). These data are currently under embargo and will be released to the public upon publication of this article or one year after submission. Access may be granted earlier upon reasonable request to the corresponding author.

## Supplementary Materials

The following supporting information can be downloaded at https://www.mdpi.com/article/10.3390/pathogens14111182/s1, Table S1: *Borrelia garinii* plasmids carrying *vlsE* gene and/or *vls* silent cassettes identified through genome annotation. References cited in Supplementary Materials [52,53].

## Abbreviations

The following abbreviations are used in this manuscript:

WGS	Whole-genome sequencing
NCBI	National Center for Biotechnology Information
MLST	Multilocus sequence typing
PacBio	Pacific Biosciences
ST	Sequence type
ANI	Average nucleotide identity
bp	Base pair
CDS	Coding sequence
SNP	Single-nucleotide polymorphism
