# Supplementary material for: Comparative Genomics of Two Newly Sequenced Rodent-Derived and One Previously Reported Tick-Derived Borrelia garinii Strains from South Korea Reveals Plasmid Variation and Virulence Gene Diversity
Source: Pathogens. 2025 Nov 18;14(11):1182. doi: 10.3390/pathogens14111182 (PMC12655509; doi:10.3390/pathogens14111182)
Supplement: Supplementary file 1 [file pathogens-14-01182-s001.zip › pathogens-3986757-supplementary.pdf]

Supplementary Materials

Table S1. *Borrelia garinii* plasmids carrying *vlsE* gene and/or *vls* silent cassettes identified through genome annotation.

Strain	Plasmid	Accession No.	Start (bp)	End (bp)	Strand	Gene Annotation (Prokka) [a]	InterProScan Repeat Count [b]
HN13	lp28-3	Current study	5846	6946	-	hypothetical protein	2
			6952	8118	-	hypothetical protein	2
			8124	8702	-	hypothetical protein	
			8708	9280	-	hypothetical protein	
			9286	9882	-	hypothetical protein	
			9827	10441	-	hypothetical protein	
			10447	11028	-	hypothetical protein	
			11034	13199	-	hypothetical protein	3
			14130	15221	+	**Variable large protein 21**	
HN18	lp28-3	Current study	92	1192	-	**Variable large protein 21**	
			1583	4147	+	hypothetical protein	7
			4276	6558	+	hypothetical protein	5
			6652	10503	+	hypothetical protein	8
	lp28-4	Current study	27857	29038	+	hypothetical protein	
935				N.D.			
BgVir				N.D.			
20047	lp36	CP028865.1	5166	6350	+	hypothetical protein	
			23136	24203	-	**Variable large protein 21**	
	lp28-3	CP028870.1	17735	22774	-	hypothetical protein	10
			22810	22989	-	hypothetical protein	
			23121	25673	-	hypothetical protein	8
	lp28-4	CP028863.1	24389	25072	+	hypothetical protein	
			25035	25571	+	hypothetical protein	
CIP103362	lp36	CP161033.1	5166	6350	+	hypothetical protein	
			23136	24203	-	**Variable large protein 21**	
	lp28-4	CP161032.1	24390	25073	+	hypothetical protein	
			25036	25572	+	hypothetical protein	

PBes	p_lp28-3	CP119351.1	203	1315	-	**Variable large protein 21**	
			1745	3478	+	hypothetical protein	4
			3435	8309	+	hypothetical protein	8
NMJW1				No plasmid			
SZ				No plasmid			
17-63N1	lp28-9	CP117844.1	130	1218	-	**Variable large protein 21**	
		CP117844.1	1612	6756	+	hypothetical protein	9
		CP117844.1	6749	12169	+	hypothetical protein	10
		CP117844.1	12162	13688	+	hypothetical protein	3
		CP117844.1	13666	13875	+	hypothetical protein	
		CP117844.1	13872	15056	+	hypothetical protein	3
	plasmid (no name)	CP117845.1	12477	12788	+	hypothetical protein	
		CP117845.1	12739	13551	+	hypothetical protein	
		CP117845.1	32256	32681	-	hypothetical protein	
		CP117845.1	33016	33327	-	hypothetical protein	
NG-Z6	lp28-3	CP117915.1	61	447	+	hypothetical protein	
FNG-1Z1	lp28-9	CP119362.1	15924	16232	-	hypothetical protein	
		CP119362.1	16386	16814	-	hypothetical protein	
17-29Z1	lp28-9	CP119378.1	15924	16232	-	hypothetical protein	
		CP119378.1	16386	16541	-	hypothetical protein	
17-54Z3	lp28-3	CP117898.1	17711	18226	-	hypothetical protein	
		CP117898.1	18207	18431	-	hypothetical protein	
		CP117898.1	18428	19843	-	hypothetical protein	
17-59N1	lp28-9	CP119387.1	15924	16232	-	hypothetical protein	
		CP119387.1	16386	16814	-	hypothetical protein	
		CP119387.1	16820	17053	-	hypothetical protein	
17-58N4	lp28-9	CP118302.1	15924	16232	-	hypothetical protein	
		CP118302.1	16386	17003	-	hypothetical protein	
FNG-2Z14	lp28-3	CP117891.1	20964	21479	-	hypothetical protein	
		CP117891.1	21460	21684	-	hypothetical protein	
		CP117891.1	21681	24245	-	hypothetical protein	4
		CP117891.1	24242	26251	-	hypothetical protein	3

[a] Product names of annotated genes were determined using the Prokka annotation pipeline [52]; [b] The number of repeated 'variable surface antigen *VlsE*' motifs was obtained from the 'Signature Description' section of each annotated gene identified by InterProScan [53]; N.D.: Not detected.
